# External Beam Breast and Chest Wall Re-Irradiation Following Resection of Ipsilateral Breast Cancer Recurrence: Dose–Toxicity Relationships and Long-Term Oncologic Outcomes

**DOI:** 10.3390/cancers18172878

**Published:** 2026-09-05

**Authors:** Suman Ghosh, Hany Soliman, Edward Chow, Hanbo Chen, Eric Leung, Irene Karam, Eileen Rakovitch, Amir H. Safavi, Danny Vesprini, Gregory J. Czarnota

**Affiliations:** 1 Department of Radiation Oncology, Sunnybrook Health Sciences Centre, University of Toronto, Toronto, ON M4N 3M5, Canada; suman.ghosh@sunnybrook.ca (S.G.); hany.soliman@sunnybrook.ca (H.S.); edward.chow@sunnybrook.ca (E.C.); hanbo.chen@sunnybrook.ca (H.C.); eric.leung@sunnybrook.ca (E.L.); irene.karam@sunnybrook.ca (I.K.); eileen.rakovitch@sunnybrook.ca (E.R.); amir.safavi@sunnybrook.ca (A.H.S.); danny.vesprini@sunnybrook.ca (D.V.); 2 Physical Sciences, Sunnybrook Research Institute, Toronto, ON M4N 3M5, Canada

**Keywords:** reirradiation, recurrent breast cancer, toxicity, external beam reirradiation

## Abstract

**Background/Objectives**: Breast/chest wall (B/CW) re-irradiation (reRT) is approached cautiously because of concerns regarding late toxicity and uncertain benefit. Contemporary data on mature toxicity and oncologic outcomes after external beam photon reRT are limited. **Methods**: This retrospective cohort included 113 consecutive patients who completed reRT after resection of locoregionally recurrent breast cancer between 2010 and 2024. The primary endpoint was cumulative incidence of late grade ≥ 2 CTCAE toxicity, analyzed using competing-risks methods. Secondary endpoints were locoregional recurrence-free survival (LRRFS) and local failure (LF), assessed using Kaplan–Meier, Cox regression, and Fine–Gray modeling. **Results**: Median age at reRT was 66 years. Most recurrences were early-stage (72.6%) and hormone receptor-positive (68.1%). Median cumulative EQD2 was 94.9 Gy, and 25.7% received IMRT. At 67.1 months’ median follow-up, 5-year cumulative incidences of grade ≥ 2 and grade 3 toxicity were 35.4% (95% CI, 25.6–45.1) and 3.9% (95% CI, 0.1–7.8), respectively. Higher cumulative EQD2 predicted grade ≥ 2 toxicity (SHR, 1.94 per 10 Gy; *p* < 0.001) and grade 3 toxicity (SHR, 4.32 per 10 Gy; *p* < 0.001). Longer interval between radiotherapy courses (*p* = 0.029) and IMRT (SHR, 0.40; *p* = 0.005) were associated with lower grade ≥ 2 toxicity. Five-year LRRFS was 84.2% (95% CI, 76.7–92.4), and LF was 6.8%. Advanced-stage disease and second or subsequent recurrence predicted inferior LRRFS. Partial B/CW reRT did not compromise disease control in early-stage recurrences. **Conclusions**: Adjuvant breast and chest wall re-irradiation using external photons achieved durable locoregional control with low rates of severe late toxicity in one of the largest reported cohorts. These findings support individualized curative-intent reRT guided by cumulative dose, technique, and recurrence risk.

## Simple Summary

Despite improvements in breast cancer treatment, cancer can still return in the same breast or chest wall in 3–15% of patients in the long term. Treating the area with radiation again may improve local control, but it is often avoided because of concerns about long-term side effects and uncertainty about potential benefit. This study evaluates outcomes in patients who received a second course of external beam radiotherapy after surgery for recurrent breast cancer. The authors aim to measure late treatment-related toxicity, identify factors associated with higher risk, and assess how effectively re-irradiation prevents further local or regional recurrence. By reporting outcomes from a large contemporary cohort with long follow-up, this research may help clinicians balance the benefits and harms of repeat radiotherapy. The findings may also guide patient selection, treatment planning, and dose decisions, while providing a benchmark for future prospective studies and the development of safer, more personalized re-irradiation approaches.

## 1. Introduction

Breast cancer is the most commonly diagnosed malignancy among women worldwide [1], and breast-conserving therapy (lumpectomy and radiation) or mastectomy with or without radiotherapy (RT) remain standard local treatment approaches [2,3,4]. Despite substantial improvements in multimodality management, ipsilateral breast or chest wall recurrence continues to occur in 3–15% of patients at long-term follow-up, depending on age, primary stage and subtypes [3,5,6]. As survivorship after primary breast cancer continues to improve [7], recurrent or new primary breast cancer arising in previously irradiated tissue is increasingly encountered in routine practice.

Historically, salvage mastectomy has been considered the standard treatment for ipsilateral recurrence after prior RT [8] because repeat breast-conserving surgery alone is associated with high local failure rates [9,10,11]. Increasing evidence now supports repeat breast conservation with re-irradiation (reRT) as an oncologically acceptable alternative in selected patients [12,13,14], and reRT after salvage mastectomy may also improve outcomes in high-risk patients with locally advanced recurrence [15,16]. For appropriately selected patients, repeat breast conservation may also preserve cosmesis, avoid the greater surgical burden of mastectomy, and reduce its potential adverse effects on body image and psychosocial well-being [17,18].

The major historical barrier to breast or chest wall reRT has been concern regarding cumulative normal-tissue toxicity in previously irradiated skin, subcutaneous tissue, chest wall, lung, heart, and regional structures [19]. This concern has contributed to inconsistent adoption of reRT, particularly outside highly selected partial-breast settings [12,20]. The existing literature is also heterogeneous with respect to re-irradiation modality and reflects geographic practice patterns [19]. The most mature long-term outcomes originate from European multicatheter interstitial brachytherapy series [21,22], whereas contemporary proton reRT data are emerging primarily from the United States [23,24]. In contrast, external beam photon reRT, despite being the most widely accessible modality worldwide, remains comparatively understudied, particularly with respect to long-term toxicity, dose–response relationships, and modifiable treatment-related factors that may mitigate morbidity. Questions also remain regarding the optimal re-irradiation dose required to achieve durable local control in the setting of presumed radioresistance of recurrent disease [25], as well as the potential impact of modern conformal techniques on toxicity outcomes. To address these knowledge gaps, we evaluated long-term oncologic and toxicity outcomes following breast or chest wall re-irradiation with external beam radiation, with specific emphasis on cumulative dose, treatment technique, and recurrence-related factors as predictors of toxicity and disease control.

## 2. Materials and Methods

### 2.1. Study Design and Patient Selection

We performed a retrospective single-institution cohort study of patients undergoing external beam re-irradiation (reRT) to the ipsilateral breast or chest wall following resection of recurrent breast cancer between January 2010 and December 2024. Institutional research ethics board approval was obtained prior to study initiation (REB project identifier number 2406).

Both in-breast recurrences after breast-conserving therapy and chest wall recurrences after mastectomy were included. Eligible patients had histologically confirmed recurrent invasive carcinoma or ductal carcinoma in situ (DCIS), complete pathological assessment of the recurrence specimen, and received curative-intent external beam re-irradiation following surgery. Exclusion criteria included unavailable prior dose-prescription data for the initial radiotherapy course, incompletely overlapping or non-overlapping reRT portals relative to the primary treatment volume, presence of macroscopic disease, and non-curative treatment intent. Patients treated with definitive-intent reRT for gross residual or unresectable disease were analyzed separately as an exploratory cohort for Grade 3 toxicity analyses.

### 2.2. Radiotherapy Details

Radiotherapy technique, target volume selection, and fractionation were determined at physician discretion according to institutional practice patterns during the study period. Treatment approaches evolved throughout the study period. All patients underwent CT-based simulation and treatment planning using either 3DCRT or tangential IMRT. For partial breast reRT, clinical target volume (CTV) delineation generally followed the NRG/RTOG 1014 approach [26], consisting of a 15 mm expansion around the lumpectomy cavity excluding the chest wall and extending no closer than 5 mm from the skin surface. Planning objectives for organs at risk followed the ALARA principle. Ipsilateral lung and heart constraints were applied to the reRT course only, including ipsilateral lung V20 < 25%, V5 < 35%, mean heart dose < 2 Gy for right-sided treatment and <3 Gy for left-sided treatment. For spinal canal and brachial plexus, cumulative Dmax constraints across both courses were applied, using 45 Gy and 66 Gy, respectively; when nodal fields were treated with historical 2D plans, the full prescription dose was conservatively assumed for these structures.

### 2.3. Data Collection

Clinical, pathological, dosimetric, and treatment-related data were abstracted from a prospectively maintained electronic medical record and supplemented by external chart review when available. Late toxicities were determined through detailed review of physician and nursing documentation, toxicity grading records, and all available radiologic investigations.

### 2.4. Endpoints

The primary endpoint was late Grade ≥ 2 toxicity according to Common Terminology Criteria for Adverse Events (CTCAE) v5.0, assessed from 3-month post-completion of reRT onward. Grade 3 toxicity was evaluated as a secondary toxicity endpoint.

Oncologic endpoints included locoregional recurrence-free survival (LRRFS) and cumulative incidence of local failure (LF). LRRFS was defined from completion of reRT to locoregional recurrence or breast cancer-related death.

### 2.5. Statistical Analysis

Descriptive statistics were summarized using medians with interquartile ranges (IQR) for continuous variables and frequencies with percentages for categorical variables.

LRRFS was estimated using the Kaplan–Meier method and compared using log-rank testing. Univariable and multivariable Cox proportional hazards regression analyses were performed to evaluate prognostic factors associated with LRRFS. Proportional hazards assumptions were assessed using Schoenfeld residual testing.

Cumulative incidences of local failure and toxicity outcomes were estimated using competing-risks methodology. For toxicity analyses, both death and local recurrence were treated as competing events. Gray’s test was used for univariable comparisons between groups, and Fine–Gray subdistribution hazards regression models were used for multivariable competing-risks analyses.

Because the study evaluated the impact of cumulative reRT dose on both oncologic outcomes and toxicity, equivalent dose in 2 Gy fractions (EQD2) was calculated using an α/β ratio of 10 Gy to provide a consistent dose scale across prescription regimens. This approach was selected to avoid over-weighting fraction size in the dose metric itself, as fractionation was evaluated separately as an independent treatment-related variable. Cumulative EQD2 was calculated by summing the initial radiotherapy and re-irradiation courses and was modeled per 10 Gy increase. Sensitivity analyses recalculated cumulative EQD2 using an α/β ratio of 3 Gy for toxicity outcomes. Radiation-related predictors, including cumulative dose, fractionation, treatment technique, and target volume, were evaluated alongside clinical and recurrence-related factors in models of toxicity and disease control. Model-based estimates were used to identify cumulative EQD2 values associated with selected predicted toxicity probabilities. An exploratory analysis was performed in patients treated with definitive-intent reRT for gross invasive disease to further evaluate Grade 3 toxicity dose–response relationships in a higher-risk population.

All statistical tests were two-sided, with *p* < 0.05 considered statistically significant. Statistical analyses were performed using R version 4.6.1 (R Foundation for Statistical Computing, Vienna, Austria).

## 3. Results

### 3.1. Primary Patient and Treatment Characteristics

The primary study cohort comprised 113 patients (descriptive parameters detailed in Table 1). The median age at re-irradiation was 66 years (IQR, 60–74). Most recurrences were invasive ductal carcinoma (84%) and hormone receptor-positive (69%). Overall, 23% of patients had locally advanced locoregional recurrence at presentation, while five patients had synchronous limited-burden asymptomatic metastatic disease identified on staging investigations but were still treated with curative-intent re-irradiation.

Surgical management at recurrence included lumpectomy in 55%, mastectomy in 31%, and local chest wall excision after prior mastectomy in 12.4%. Among the 35 patients undergoing salvage mastectomy, indications for re-irradiation included locally advanced recurrence (n = 24), margin-positive disease (n = 3), involved sentinel node (n = 2), or the presence of ≥ 2 high-risk features, including young age, lymphovascular invasion, grade 3 histology, or multifocal disease (n = 8). Regional nodal irradiation (RNI) was delivered in 21 patients, primarily for nodal recurrence or high-risk features without axillary staging. No patient received repeat regional nodal irradiation. Among 80 node-negative early-stage recurrences, 27 patients underwent partial breast or partial chest wall re-irradiation. Median interval between radiotherapy courses was 12.3 years (IQR, 7.2–17.8) with a median cumulative EQD2_α/β=10_ of 94.9 Gy (IQR, 92.3–104.3).

The median prescribed re-irradiation dose was 45 Gy (IQR, 42.5–45), with surgical bed boost delivered in 11 patients. Patients with R1 resection received significantly higher cumulative tumor-bed dose compared with R0 resections (median EQD2 50 Gy vs. 44.3 Gy; *p* < 0.001); however, prescription dose did not significantly vary by stage (*p* = 0.516), recurrence timing (*p* = 0.158), or grade (*p* = 0.709). The most common regimen was 45 Gy in 25 fractions (44.2%), while moderate hypofractionation (40 Gy in 15 fractions) was used in 32% of patients. The majority were treated with 3DCRT, while tangential IMRT was utilized in 25.7%. IMRT was used more frequently earlier in the study period, when physicians had greater concern regarding toxicity; median follow-up was 115.6 months for IMRT and 52.9 months for 3DCRT.

Adjuvant systemic therapy was individualized according to recurrence stage, patient age, and patient preference. Overall, 46 patients (40.7%) received adjuvant chemotherapy, 72 received endocrine therapy, 14 received HER2-targeted therapy, and 12 received no adjuvant systemic therapy.

The secondary exploratory cohort included 36 patients who were treated with reRT without surgery for symptomatic locoregional recurrence (Supplementary Table S1). The median age was 69.5 years (IQR, 58.5–81.8). Thirty-one patients (86%) had metastatic disease at the time of re-irradiation, of whom 11 had controlled distant disease. Twenty-seven patients (75%) had T4 locoregional disease with skin and/or chest wall involvement. Prescription doses ranged from 30 Gy in five fractions to 70 Gy in 35 fractions. The median interval between radiotherapy courses was 32.9 months (IQR, 20.2–96.6), and the median cumulative EQD2 was 99.8 Gy (IQR, 90.3–115.7).

### 3.2. Oncologic Efficacy

At a median follow-up of 67.1 months (IQR, 52.5–81.8), five-year LRRFS was 84.2% (95% CI, 76.7–92.4), overall survival (OS) was 82.1% (95% CI, 74.1–91.0), and the 5-year cumulative incidence of local failure (LF) was 6.8%. Among seven local failure events, four occurred with synchronous metastatic progression, and three were isolated local recurrences. All 16 breast cancer-related deaths occurred in the setting of distant metastatic events. In non-metastatic patients (n = 108), the corresponding LRRFS and OS were 85.9% (95% CI, 78.6–93.9) and 84.2% (95% CI, 76.5–92.7), respectively.

**Figure 1:**
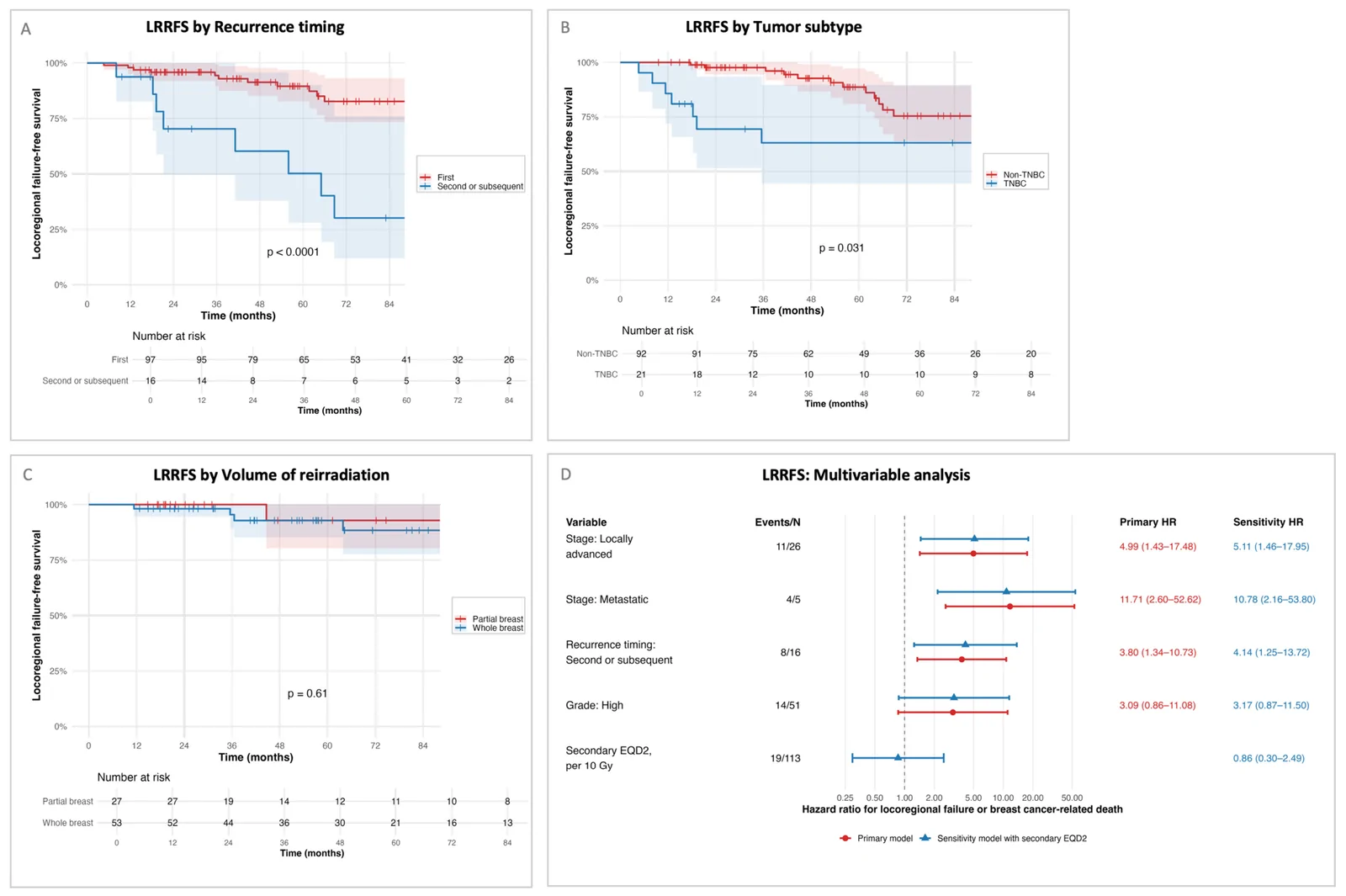
Oncologic outcomes after adjuvant breast/chest wall re-irradiation. (**A**) Locoregional recurrence-free survival (LRRFS) according to recurrence timing (first versus second/subsequent recurrence). (**B**) LRRFS according to tumor subtype (TNBC versus non-TNBC). (**C**) LRRFS according to treatment volume (partial versus whole-breast/chest wall re-irradiation) among patients with early-stage recurrences. (**D**) Multivariable Cox regression analysis for LRRFS stratified by TNBC status. Hazard ratios are shown with 95% confidence intervals.

On univariable analyses (Table 2, Figure 1A–C), inferior LRRFS was associated with advanced stage (global *p* < 0.001), high grade (*p* = 0.013), second or subsequent recurrence (*p* < 0.001), dermal lymphovascular invasion (LVI; *p* < 0.001), and triple-negative subtype (*p* = 0.03). Correspondingly, cumulative incidence of local failure was significantly higher among patients with dermal LVI (*p* = 0.003), second or subsequent recurrence (*p* = 0.013), and high-grade disease (*p* = 0.034), with a trend toward higher incidence in TNBC (*p* = 0.07). Predictors of LRRFS also similarly influenced OS (Supplementary Table S2). Adjuvant chemotherapy was not associated with LRRFS on univariable analysis (*p* = 0.26), consistent with treatment-by-indication effects.

The proportional hazards assumption was violated for TNBC status (*p* = 0.002), indicating a time-dependent effect with substantially higher early LRRFS hazard that attenuated over time; therefore, multivariable Cox regression was stratified by TNBC status. In this model, locally advanced disease (HR 4.99, 95% CI 1.43–17.5; *p* = 0.012), metastatic disease (HR 11.71, 95% CI 2.6–52.6; *p* = 0.001), and second or subsequent recurrence (HR 3.8, 95% CI 1.34–10.7; *p* = 0.012) were independently associated with inferior LRRFS (Figure 1D). On multivariable Fine–Gray regression, dermal LVI (absence of dermal LVI: SHR 0.21, 95% CI 0.06–0.75; *p* = 0.016) and second or subsequent recurrence (SHR 6.24, 95% CI 1.3–29.9; *p* = 0.02) were independently associated with higher local failure incidence, while high-grade disease demonstrated a strong trend (SHR 9.96, 95% CI 0.78–126.2; *p* = 0.07).

No significant differences were observed between R0 and R1 resection status for LRRFS (*p* = 0.87) or local failure (*p* = 0.96), although higher cumulative EQD2 was delivered in R1 resections. Similarly, among patients with early-stage recurrences, partial breast or chest wall re-irradiation demonstrated no difference in LRRFS (*p* = 0.61) or local failure incidence (*p* = 0.26) compared with whole-breast irradiation. Partial- and whole-breast/chest wall re-irradiation groups were broadly similar with respect to LRRFS-related disease factors, including recurrence timing, grade, dermal/LVI status, and subtype, with differences mainly observed in age and secondary EQD2 distribution despite similar median dose (Supplementary Table S3). Notably, re-irradiation dose itself was not associated with LRRFS in either univariable (HR per 10 Gy EQD2 increase 1.25, 95% CI 0.61–2.57; *p* = 0.54) or sensitivity multivariable analyses (HR 0.86, 95% CI 0.30–2.49; *p* = 0.78) (Figure 1D, Supplementary Table S4).

### 3.3. Toxicity Outcomes

The 5-year cumulative incidence of Grade ≥ 2 and Grade 3 toxicity was 35.4% (95% CI 25.6–45.1) and 3.9% (95% CI 0.1–7.8), respectively.

The most frequent Grade ≥ 2 toxicity involved subcutaneous and soft tissue effects (fibrosis, breast discomfort and chronic ulcer in 34.5%). Rib fracture was incidentally identified in 19 patients (grade I), although only six patients required analgesia for symptomatic pain (Supplementary Table S5). Other Grade ≥ 2 toxicities included pneumonitis (5.3%), lymphedema (1.8%), shoulder dysfunction (1.8%), and one event of cardiomyopathy. Among four patients with tissue expanders following mastectomy, two required surgical management (Grade 3): one required expander removal after soft tissue infection involving a latissimus dorsi flap and tissue expander, followed by further reconstructive procedures for fibrotic scarring; the second required capsulectomy for tight capsular contracture. One patient developed Grade 2 contracture not requiring intervention, and one had an uncomplicated cosmetic outcome. There was no Grade 4 or 5 toxicity event.

**Figure 2:**
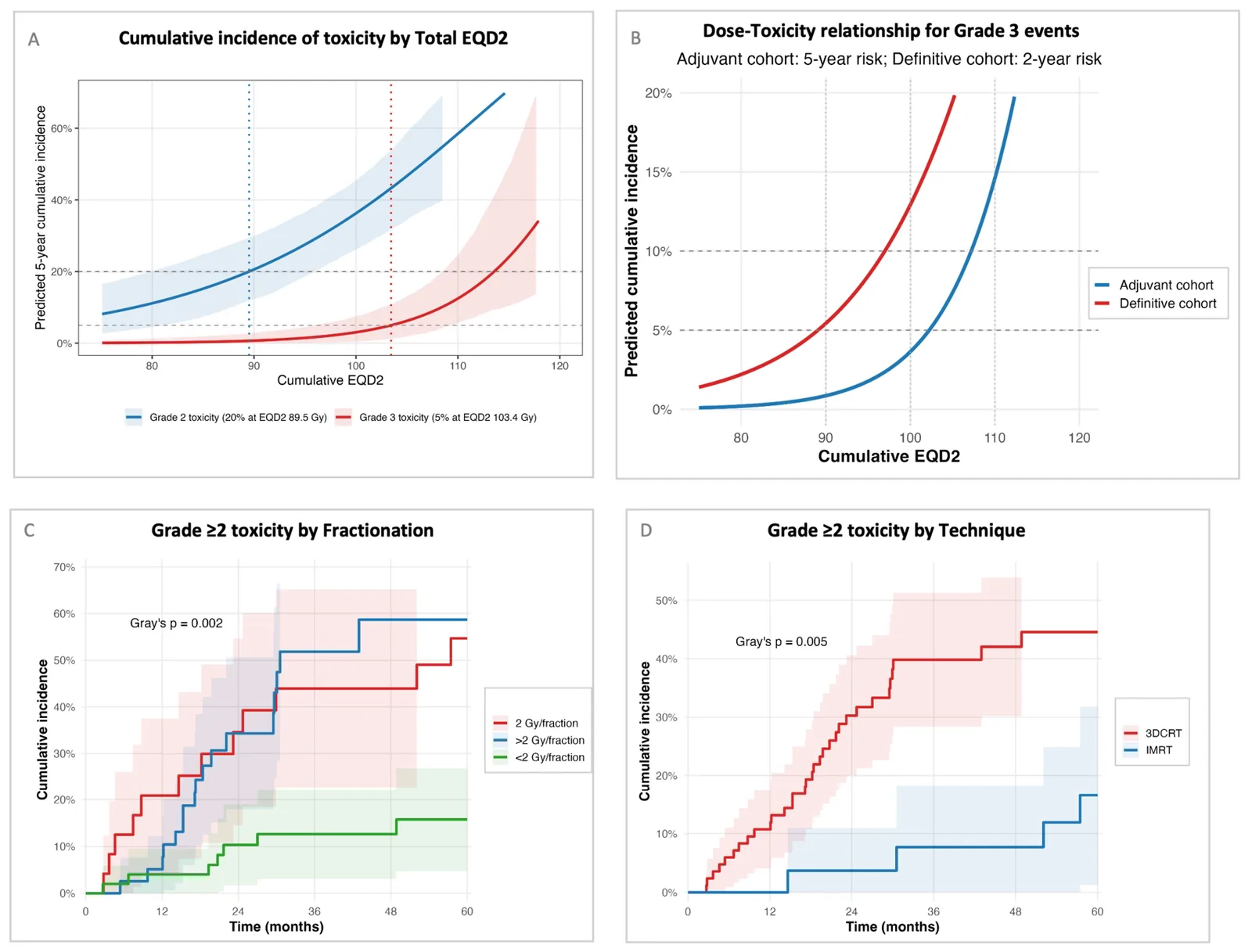
Univariate analysis of dose–toxicity relationships and cumulative incidence of Grade ≥ 2 toxicity. (**A**) Predicted 5-year cumulative incidence of Grade ≥ 2 and Grade 3 toxicity according to cumulative EQD2. Vertical dashed lines indicate the cumulative EQD2 associated with a predicted 20% risk of Grade ≥ 2 toxicity (88.4 Gy) and a 5% risk of Grade 3 toxicity (102.2 Gy). (**B**) Predicted Grade 3 toxicity according to cumulative EQD2 in the adjuvant and definitive re-irradiation cohorts. (**C**) Cumulative incidence of Grade ≥ 2 toxicity according to fractionation regimen. (**D**) Cumulative incidence of Grade ≥ 2 toxicity according to radiation technique (3DCRT versus IMRT).

On univariable competing-risks analyses, cumulative dose, interval between radiotherapy courses, treatment technique, and fractionation were significantly associated with Grade ≥ 2 toxicity (Table 3, Figure 2A,C,D). At 5 years, IMRT demonstrated substantially lower Grade ≥ 2 toxicity compared with 3DCRT (15% vs. 43.4%; Gray’s *p* = 0.004). Similarly, lower dose per fraction (<2 Gy) was associated with lower toxicity (15.3%) compared with conventional or hypofractionated regimens (52.4–55.8%; Gray’s *p* = 0.004).

**Figure 3:**
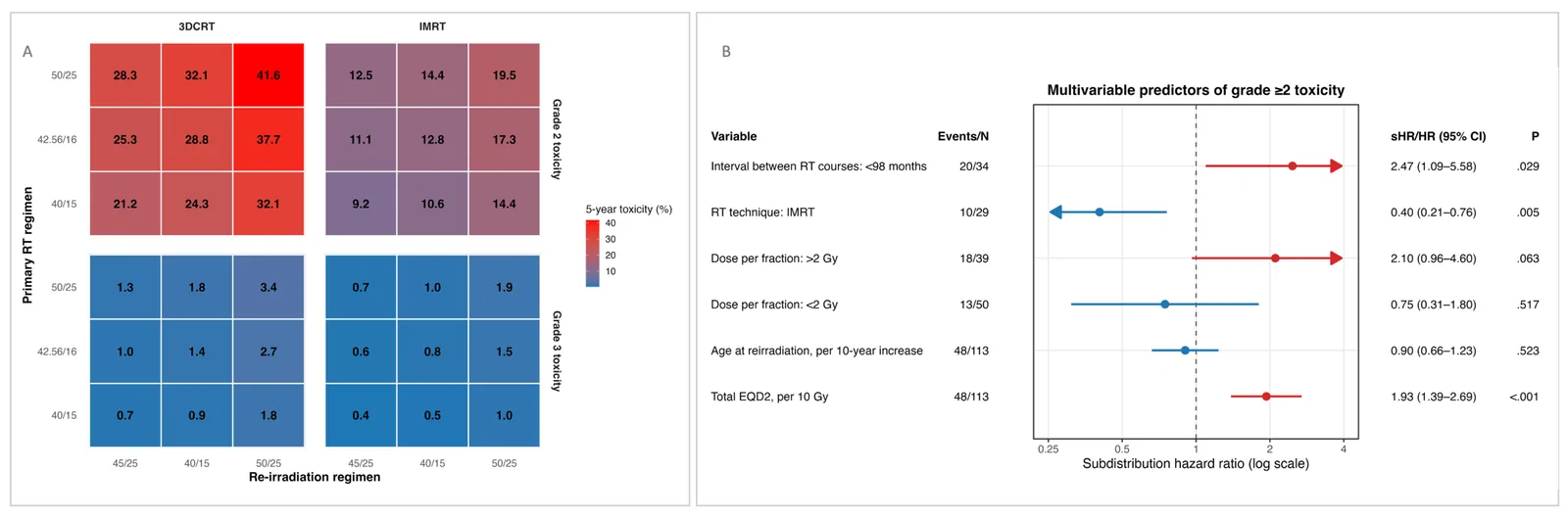
Multivariable predictors and model-based estimates of toxicity. (**A**) Predicted cumulative incidence of Grade ≥ 2 and Grade 3 toxicity according to primary and re-irradiation dose-fractionation schedules and radiation technique. (**B**) Multivariable Fine–Gray regression analysis for Grade ≥ 2 toxicity. Subdistribution hazard ratios (sHRs) are shown with 95% confidence intervals.

On multivariable Fine–Gray regression, cumulative EQD2 remained the strongest independent predictor of Grade ≥ 2 toxicity (SHR 1.94 per 10 Gy increase, 95% CI 1.39–2.70; *p* < 0.001). Longer interval between radiotherapy courses (*p* = 0.029) and IMRT (SHR 0.4, 95% CI 0.21–0.76, *p* = 0.005) remained independently associated with lower toxicity. Hypofractionation demonstrated a borderline association with increased toxicity (SHR 2.1, 95% CI 0.96–4.6; *p* = 0.06); however, 1.8 Gy/fraction had no impact (*p* = 0.52) after multivariable adjustment. Based on model estimates, the cumulative EQD2 required to maintain predicted 5-year Grade ≥ 2 toxicity below 20% was approximately 83 Gy (95% CI, 72.3–91.1) for 3DCRT and 98 Gy (95% CI, 89.6–109.6) for IMRT (Figure 3). Age at re-irradiation was correlated with interval between radiotherapy courses (Spearman ρ = 0.45, *p* < 0.001), and the univariable association between age and Grade ≥ 2 toxicity was attenuated after adjustment for interval. Sensitivity analyses using cumulative EQD2 α/β = 3 showed similar directional estimates; the univariable sHR for cumulative EQD2 α/β = 3 was 2.57 per 10 Gy increase (95% CI, 1.40–4.72; *p* = 0.002), with detailed results provided in Supplementary Table S6.

Grade 3 toxicity remained uncommon overall and was not significantly associated with interval, technique, or fractionation (Supplementary Table S7). A total of six Grade 3 events were observed, including one radiation pneumonitis requiring admission, two late skin ulcers requiring local wound intervention, one rib fracture requiring intravenous analgesics, and two tissue-expander-related complications requiring surgical management. Cumulative EQD2 was the only significant predictor in univariate analysis, with a 5% predicted risk at approximately 102 Gy. Beyond this threshold, the dose–toxicity curve demonstrated a steep increase in predicted Grade 3 risk (SHR 4.32 per 10 Gy increase, 95% CI 2.03–9.2; *p* < 0.001).

#### Grade 3 Toxicity and Outcomes in the Secondary Exploratory Cohort with Gross Disease

Because Grade 3 toxicity events were infrequent in the adjuvant cohort, exploratory analyses were performed in 36 patients treated with definitive-dose re-irradiation for gross invasive locoregional recurrence. The cumulative incidence of Grade 3 or higher toxicity in this secondary cohort at 1 and 2 years was 13.5% (95% CI 1.0–26.3) and 18.3% (95% CI 3.1–33.6), respectively. Fine–Gray modeling demonstrated a similarly steep dose–toxicity relationship, with cumulative EQD2 independently associated with Grade 3 toxicity (SHR 2.51 per 10 Gy increase, 95% CI 1.35–4.66; *p* = 0.003). Compared with the adjuvant cohort, the dose–response curve demonstrated a leftward shift with lower cumulative EQD2 thresholds for Grade 3 toxicity while maintaining a steep dose-dependent pattern (Figure 2B). Grade 3 toxicity at 2 years was numerically higher with 3DCRT compared with IMRT (32.1% vs. 10.9%), although this difference was not statistically significant (*p* = 0.11). In this cohort, 1- and 2-year locoregional recurrence-free survival were 62.9% (95% CI, 48.7–81.1) and 24.9% (95% CI, 13.9–44.7), respectively. The corresponding cumulative incidences of local failure were 18.7% (95% CI, 4.9–32.2) and 33.9% (95% CI, 13.7–54.1), respectively. The only factor significantly associated with locoregional recurrence-free survival was pre-re-irradiation distant disease status, with progressive distant disease associated with inferior outcomes (HR, 5.52; 95% CI, 1.60–19.1; *p* = 0.006).

## 4. Discussion

In this contemporary cohort of patients undergoing breast or chest wall re-irradiation using external beam photons, we demonstrate favorable long-term oncologic outcomes with acceptable rates of severe toxicity. Importantly, cumulative dose emerged as the dominant determinant of late toxicity, while treatment interval and radiation technique independently modified risk. These findings provide clinically actionable information in a setting where mature long-term external photon reRT data remain limited, and practice variation remains substantial.

Existing evidence for external beam photon re-irradiation has predominantly focused on partial breast reRT in highly selected early-stage recurrences [19,27]. Consistent with the prospective data [20], our results support the oncologic safety of partial breast or limited chest wall reRT in appropriately selected early-stage recurrences, with excellent local control and low rates of severe toxicity. Because the present cohort included patients treated since 2010, predating prospective external photon partial breast reRT studies, a proportion of favorable early-stage recurrences received whole-breast or chest wall reRT. This provided a clinically informative cohort demonstrating that whole-breast/chest wall reRT may also be feasible in patients unsuitable for limited-volume treatment, including those with locally advanced or biologically high-risk disease. These findings additionally contextualize the evolving global adoption of breast reRT, where recent practice-pattern surveys demonstrate increasing utilization and growing clinician confidence in re-irradiation strategies [28].

The oncologic outcomes observed in the present study compare favorably with published re-irradiation series [27,29,30,31]. Despite inclusion of >25% of patients with locally advanced recurrence, 5-year LRRFS exceeded 84%, and the cumulative incidence of local failure was only 6.8%. These outcomes were comparable to the NRG/RTOG 1014 trial, which reported 5% local failure despite restricting eligibility to highly selected early-stage node-negative recurrences [20]. Approximately 90% of patients with a first recurrence remained alive and recurrence-free at 5 years, whereas outcomes were substantially worse after second or subsequent recurrences, underscoring the importance of effective salvage treatment at first relapse.

The principal finding of this study is the strong dose–toxicity relationship observed with breast reRT. Cumulative dose was independently associated with both Grade ≥ 2 and Grade 3 toxicity. For Grade ≥ 2 toxicity, each 10 Gy increase in cumulative EQD2 increased risk by approximately 80–90%, with toxicity rising progressively beyond cumulative EQD2 levels of 90–95 Gy. Lower dose-per-fraction regimens (<2 Gy/fraction) were associated with substantially lower absolute rates of Grade ≥ 2 toxicity compared with conventional or hypofractionated regimens, although this association attenuated after adjustment for cumulative dose and treatment-related factors. On the contrary, a statistical trend toward increased toxicity with hypofractionation was observed in multivariable analysis. Interestingly, re-irradiation dose was not associated with improved LRRFS or local control in this analysis; however, confounding by indication cannot be excluded, as higher doses were often used in higher-risk settings (e.g., R1 resections). Therefore, while cumulative dose was strongly associated with toxicity, these data do not establish whether dose escalation improves oncologic outcomes, and they support careful individualization of dose beyond the standard adjuvant regimen.

Toxicity was also strongly influenced by treatment technique. Across all cumulative dose ranges, IMRT was associated with substantially lower predicted toxicity than 3DCRT, likely reflecting improved dose homogeneity and reduced overlap of intermediate-dose regions. This association should be interpreted in the context of non-randomized treatment selection and temporal changes in practice, as technique choice may have been influenced by physician concern regarding anticipated toxicity. Similarly, the inverse association between interval from prior radiotherapy and toxicity is biologically plausible [25] but may also reflect differences in recurrence biology, prior treatment burden, and patient selection. Overall, the present data provide clinically relevant dose estimates and suggest that toxicity risk remains modifiable by dose, technique and possibly fractionation.

Late Grade 3 events were infrequent, with a 5-year cumulative incidence of 3.9%, and the overall toxicity profile fares well with the reported series [19,20,31,32,33]. Importantly, for Grade 3 toxicity, the dose–response curve demonstrated a steep increase beyond approximately 105 Gy cumulative EQD2. This pattern was reproduced in an exploratory cohort of patients treated with definitive-intent re-irradiation for gross invasive recurrence. In the definitive cohort, the dose–response relationship also demonstrated a leftward shift, with lower cumulative EQD2 thresholds for Grade 3 toxicity while maintaining a similarly steep dose-dependent pattern. The shorter interval between RT courses and the uniformly advanced nature of disease, including universal T4 tumors with skin involvement, likely contributed to this lower threshold. In addition, treatment-related injury may have been compounded by tumor regression, resulting in non-healing ulceration or chest wall defects.

The secondary exploratory cohort suggests that definitive re-irradiation may provide meaningful local control in selected patients with gross locoregional recurrence who are not candidates for surgical management. However, outcomes in this cohort were strongly influenced by distant disease status. In particular, patients with progressive metastatic disease had limited durability of benefit, underscoring that the role of re-irradiation in this setting is closely linked to systemic disease control and should be individualized within a multidisciplinary framework.

The present study has several limitations. Its retrospective single-institution design introduces potential selection bias, particularly regarding treatment technique, dose, and fractionation selection. The primary cohort was clinically heterogeneous with respect to stage, surgical context, radiation volume, treatment era, and recurrence risk; therefore, associations involving IMRT, fractionation, interval between RT courses, and cumulative EQD2 should not be interpreted as causal. Toxicity assessment relied on retrospective documentation and may underestimate lower-grade events, such as fibrosis, pain, cosmetic change, and functional impairment. Conversely, as pre-existing morbidity was not systematically documented before re-irradiation, recorded late effects may represent cumulative morbidity after repeat local therapy rather than toxicity attributable solely to re-irradiation. Because a proportion of patients received historical 2D primary radiotherapy, cumulative deformable dosimetric reconstruction and NTCP modeling were not feasible; cumulative dose therefore reflects prescription dose rather than true organ-specific exposure. The appropriate α/β ratio for re-irradiated breast and chest wall normal tissues also remains uncertain; sensitivity analyses using α/β = 3 were performed but do not validate a single radiobiologic model. The technique-specific model-derived estimates should therefore be considered exploratory estimates rather than established safety limits or clinical dose constraints. In addition, the relatively low number of local failure and Grade 3 toxicity events resulted in wide confidence intervals for some estimates. Nevertheless, to our knowledge, this represents the largest contemporary external beam photon breast re-irradiation cohort. Beyond reporting mature oncologic and toxicity outcomes, the study provides quantitative dose–response estimates and clinically relevant predictors that may assist treatment selection, dose prescription, and patient counseling in routine practice.

## 5. Conclusions

Adjuvant breast and chest wall re-irradiation using external beam photons achieved durable locoregional control with low rates of severe late toxicity in one of the largest reported contemporary photon re-irradiation cohorts. Cumulative dose was the dominant determinant of toxicity, while IMRT was associated with lower toxicity risk. Partial breast/chest wall reRT also appeared safe in selected early-stage recurrences. These findings support individualized breast reRT strategies integrating cumulative dose, treatment volume, and modern conformal techniques to optimize the therapeutic ratio.

## Figures and Tables

**Table 1:** Descriptive details of tumor and treatment-related details.

Variable	Subgroups	Absolute Number	Percentage
Histology	IDC	95	84.1
	ILC or other histologies	16	14.2
	DCIS	2	1.8
Recurrence timing	First	97	85.8
	Second or subsequent	16	14.2
Primary (pT) stage at recurrence	Tx	2	1.8
	Tis	2	1.8
	T1	56	49.6
	T2	27	23.9
	T3	4	3.5
	T4	22	19.4
Nodal (N) stage at recurrence	pN0	54	47.8
	cN0 pNx	42	37.2
	pN1	11	9.7
	pN2	4	3.5
	pN3	2	1.8
Overall stage	Early	82	72.6
	Locally advanced	26	23
	Metastatic	5	4.4
Tumor subtype	HR + HER2-	78	69
	HER2+	14	12.4
	Triple negative	21	18.6
Lymphovascular space invasion	Present	24	21.2
	Absent	89	78.8
Dermal lymphovascular invasion (LVI)	Yes	14	12.4
	No	99	87.6
Grade	Early/Intermediate	62	54.87
	High	51	45.13
Salvage surgery for local recurrence (n = 111 ^a^)	BCS	62	55.9
	Mastectomy	35	31.5
	Local chest wall excision	14	12.6
Axillary surgery at recurrence	Sentinel biopsy	58	51.3
	Axillary dissection	11	9.7
	None	44	38.9
Margin status at recurrence surgery	Uninvolved (R0)	98	86.7
	Microscopic involved (R1)	15	13.3
Adjuvant systemic therapy	Chemotherapy	29	25.7
	Endocrine therapy (ET) alone	55	48.7
	Chemotherapy + ET	17	15
	None	12	10.6
Re-irradiation target volume	Whole breast	38	33.6
	Chest wall	27	23.9
	Partial breast/Chest wall	27	23.9
	Breast/CW + RNI	21	18.6
Re-irradiation dose per fraction	2 Gy/fraction	24	21.2
	>2 Gy/fraction	39	34.5
	<2 Gy/fraction	50	44.2
Re-irradiation technique	3DCRT	84	74.3
	IMRT	29	25.7
Linear Variable		Median	IQR
Age (Years)		66	60–74
Interval between RT courses (Years)		12.3	7.2–17.8
Cumulative EQD2 (Gy)		94.9	92.3–104.3

[IQR = interquartile range, IDC = infiltrative ductal carcinoma, ILC = infiltrative lobular carcinoma, DCIS = ductal carcinoma in situ, RNI = regional nodal irradiation, 3DCRT = 3-dimensional conformal radiotherapy, IMRT = intensity-modulated radiotherapy; ^a^ denominator for salvage local surgery is 111, as 2 patients underwent axillary lymph node dissection alone].

**Table 2:** Results of univariate analyses for locoregional recurrence-free survival (LRRFS) and cumulative incidence of local failure (LF).

Variable	Subgroups	5-Year LRRFS	*p*	5-Year CIF LF	*p*
Histology	IDC (95)	86.7 (79.1–95)	0.2	6.5 (0.8–12.3)	0.33
	ILC or other (16)	70.3 (49.3–100)		11.1 (0–32.8)	
	DCIS (2)	100		0	
Dermal LVI	Yes (n = 14)	57.1 (36.3–89.9)	**<0.001**	23.8 (0–49)	**0.003**
	No (n = 99)	88.4 (81.0–96.5)		4.8 (0–10.3)	
Recurrence timing	First (n = 97)	89.5 (82.7–96.9)	**<0.001**	4 (0–8.6)	**0.013**
	Second or subsequent (n = 16)	50.2 (28.0–90.2)		29.7 (0–62.5)	
Grade	Early/Intermediate (n = 62)	90.1 (81.2–100)	**0.013**	2.5 (0–7.4)	**0.034**
	High (n = 51)	77.4 (65.7–91.1)		12.5 (1.7–23.1)	
Stage	Early (n = 82)	93.1 (86.6–100)	**<0.001**	5.7 (0–12.2)	0.17
	Locally advanced (n = 26)	64.7 (47.4–88.3)		9.8 (0–23.5)	
	Metastatic (n = 5)	53.3 (21.4–100)		20 (0–59.2)	
Tumor Subtype	HR or HER2+ (n = 92)	88.6 (80.8–97.1)		5.03 (0–10.8)	0.077
	TNBC + (n = 21)	63.1 (44.3–89.6)	**0.03**	17.7 (0–37.7)	
Resection margin status	R0 + (n = 98)	84.1 (75.9–93.1)	0.87	6.9 (0.7–13.1)	0.96
	R1 + (n = 15)	85.1 (68–100)		8.3 (0–24.6)	
Adjuvant chemotherapy	Chemo (n = 46)	85.4 (75.1–97)	0.25	7.2 (0–15.1)	0.51
	No chemo (n = 67)	82.7 (72.1–94.9)		7.7 (0–16.6)	
Volume of reRT	Partial (n = 27)	92.9 (80.3–100)	0.61	0	0.26
	Whole (n = 53)	92.8 (85.2–100)		5.4 (0–12.8)	

[IDC = infiltrative ductal carcinoma, ILC = infiltrative lobular carcinoma, DCIS = ductal carcinoma in situ, LVI = lymphovascular invasion, TNBC = triple-negative breast cancer, reRT = re-irradiation]. Significant association are bolded.

**Table 3:** Predictors of late Grade ≥ 2 toxicity following breast re-irradiation.

Variables	Subgroup	5-Year Incidence	*p* (Gray)	UV sHR	UV *p*	MV sHR	MV *p*	
Interval between RT courses	≥98 months	26.6 (15.6–37.6)	0.021	Ref		Ref		
	<98 months	56.6 (38.4–74.8)		2.07 (1.14–3.76)	0.017	2.47 (1.09–5.58)	0.029	
RT Technique	3DCRT	43.4 (31.4–55.3)	0.004	Ref				
	IMRT	15.0 (1.1–28.9)		0.39 (0.21–0.72)	0.003	0.40 (0.21–0.76)	0.005	
Dose per fraction at re-irradiation	2 Gy	52.4 (30.9–73.9)	0.004	Ref				
	>2 Gy	55.8 (35.8–75.9)		0.91 (0.45–1.82)	0.780	2.10 (0.96–4.60)	0.063	
	<2 Gy	15.3 (4.6–26.0)		0.32 (0.16–0.65)	0.002	0.75 (0.31–1.80)	0.517	
Prior mastectomy	Yes	42.1 (27.1–57.1)	0.576	Ref		-		
	No	31.3 (18.3–44.3)		0.83 (0.47–1.47)	0.525			
Re-irradiation setting and volume	Repeat BCS + Partial RT	29.3 (8.3–50.3)	0.543	Ref		-		
	Repeat BCS + Whole breast RT	34.9 (17.0–52.7)		1.09 (0.49–2.41)	0.83			
	Post-mastectomy reRT	32.0 (14.0–49.9)		0.88 (0.36–2.18)	0.78			
	Regional nodal RT	49.2 (26.1–72.3)		1.61 (0.67–3.88)	0.28			
Age at re-irradiation	Per 10-year increase	-		0.70 (0.55–0.88)	0.003	0.90 (0.66–1.23)		0.523
Cumulative EQD2	Per 10 Gy increase	-		1.96 (1.44–2.66)	<0.001	1.93 (1.39–2.69)		<0.001

[UV = univariate Fine–Gray model, MV = multivariable Fine–Gray model, sHR = subdistribution hazard ratio, 3DCRT = 3-dimensional conformal radiotherapy, IMRT = intensity-modulated radiotherapy, BCS = breast conservation surgery].

## Data Availability

De-identified data specific to this study will be made available upon reasonable request to the corresponding author.

## Supplementary Materials

The following supporting information can be downloaded at: https://www.mdpi.com/article/10.3390/cancers18172878/s1, Table S1: Descriptive characteristics of the secondary sensitivity cohort with gross disease (N = 36). Table S2: Results of univariate analyses for 5-year overall survival. Table S3: Baseline heterogeneity in partial vs whole breast reRT among early-stage recurrences. Table S4: Predictors of locoregional recurrence-free survival (LRRFS) following breast re-irradiation in multivariable Cox regression stratified by TNBC status. Table S5: Descriptive details of CTCAE grade toxicity distributions across domains. Table S6: Sensitivity analyses of late Grade ≥ 2 toxicity following breast re-irradiation with EQD2 α/β = 3. Table S7. Predictors of late Grade 3 toxicity following breast re-irradiation on univariate analysis.

## Abbreviations

The following abbreviations are used in this manuscript:

2D	Two-dimensional
3DCRT	Three-dimensional conformal radiotherapy
ALARA	As low as reasonably achievable
B/CW	Breast/chest wall
BCS	Breast-conserving surgery
CI	Confidence interval
CIF	Cumulative incidence function
CT	Computed tomography
CTCAE	Common Terminology Criteria for Adverse Events
CTV	Clinical target volume
CW	Chest wall
DCIS	Ductal carcinoma in situ
EQD2	Equivalent dose in 2 Gy fractions
ET	Endocrine therapy
Gr	Grade
Gy	Gray
HER2	Human epidermal growth factor receptor 2
HR	Hormone receptor; hazard ratio, according to context
IDC	Invasive ductal carcinoma
ILC	Invasive lobular carcinoma
IMRT	Intensity-modulated radiotherapy
IQR	Interquartile range
LF	Local failure
LRRFS	Locoregional recurrence-free survival
LVI	Lymphovascular invasion
MV	Multivariable
NRG/RTOG	NRG Oncology/Radiation Therapy Oncology Group
NTCP	Normal tissue complication probability
OS	Overall survival
R0	Microscopically margin-negative resection
R1	Microscopically margin-positive resection
REB	Research Ethics Board
reRT	Re-irradiation
RNI	Regional nodal irradiation
RT	Radiotherapy
sHR	Subdistribution hazard ratio
TNBC	Triple-negative breast cancer
UV	Univariable
V5	Volume receiving at least 5 Gy
V20	Volume receiving at least 20 Gy
α/β	Alpha/beta ratio
