# Supplementary material for: External Beam Breast and Chest Wall Re-Irradiation Following Resection of Ipsilateral Breast Cancer Recurrence: Dose–Toxicity Relationships and Long-Term Oncologic Outcomes
Source: Cancers (Basel). 2026 Sep 5;18(17):2878. doi: 10.3390/cancers18172878 (PMC13565669; doi:10.3390/cancers18172878)
Supplement: Supplementary file 1 [file cancers-18-02878-s001.zip › cancers-4498052-supplementary.pdf]

Supplementary Table S1. Descriptive characteristics of the Secondary Sensitivity cohort with Gross Disease (N=36).

Variable	Subgroups	Absolute number	Percentage
Reirradiation dose prescription	66-70 Gy/ 33-35 fractions	3	8.4
	50-60 Gy/ 25-30 fractions	6	16.7
	30-45 Gy/ 10-15 fractions	12	33.3
	30-40 Gy/ 5 fractions	8	22.2
	65 Gy/50 fractions	7	19.4
Reirradiation Fractionation	Conventional (1.8-2.2 Gy/fraction)	7	19.4
	Moderate hypofractionation (>2.2 -5 Gy/fraction)	14	39
	Extreme hypofractionation (>5 Gy/fraction	8	22.2
	Hyperfractionation (<1.8 Gy/fraction)	7	19.4
Reirradiation technique	3DCRT	13	36.1
	IMRT	23	63.9
Local disease status at Reirradiation	Localized (T2/T3 N0)	13	36.1
	Locally advanced (T4 or N1-N3)	23	63.9
Distant disease status at Reirradiation	No metastatic disease	5	13.8
	Controlled metastatic disease	11	30.6
	Progressive metastatic disease	20	55.6
Tumor subtype	HR+ HER2 -	14	38.9
	HER2+	9	25
	Triple negative	13	36.1
**Linear Variable**		**Median**	**IQR**
Interval from prior RT Course, months		32.9	20.2- 96.6
Age, years		69	58 -81

Supplementary Table S2. Results of Univariate analyses for 5-year Overall Survival

Variable	Subgroups	5-year OS (95% CI)	p-value
Histology	IDC (95)	83.4 (75.3 – 93.3)	0.48
	ILC or other (16)	79.1 (60.5 -100)	
	DCIS (2)	100	
Dermal Lymphovascular invasion	Yes (n=14)	50.9 (28.8 – 90)	**<0.001**
	No (n=99)	86.8 (79 – 95.4)	
Recurrence timing	First (n=97)	84.6 (76.5 - 93.7)	**0.02**
	Second or subsequent (n=16)	65.8 (43.3 – 100)	
Grade	Early/Intermediate (n=62)	84.1 (73 – 96.9)	0.4
	High (n=51)	79 (67.5 – 92.4)	
Stage	Early (n=82)	89.7 (81.1 – 97.9)	**<0.001**
	Locally advanced (n=26)	69.2 (52.4 – 91.5)	
	Metastatic (n=5)	50 (18.7 – 100)	
Tumour Subtype	HR or HER2+	88.2 (80.2 – 96.9)	0.056
	Triple negative	56.5 (37.7 – 84.7)	
Resection margin status	R0	81.7 (73 – 91.5)	0.76
	R1	83.5 (64.9 – 100)	
Adjuvant chemotherapy	Chemotherapy	81.7 (70.3 – 95.1)	0.26
	No chemotherapy	82.3 (71.5 – 94.8)	

[IDC = Infiltrative ductal carcinoma, ILC= Infiltrative lobular carcinoma, DCIS= Ductal carcinoma in-situ]

Supplementary Table S3. Baseline heterogeneity in Partial vs Whole breast reRT among early stage recurrences

Variable	Partial breast/ Chest wall (N=27)	Whole breast/ Chest wall (N=53)	P
Age at reirradiation, years	64.0 (61.0–68.5)	72.0 (63.0–76.0)	**0.016**
Interval between RT courses, months	160.8 (110.4–218.0)	184.3 (109.5–239.7)	0.508
Recurrence timing: second/subsequent	3 (11.1%)	4 (7.5%)	0.683
High grade	9 (33.3%)	22 (41.5%)	0.628
Dermal/LVI: yes	0 (0.0%)	3 (5.7%)	0.547
Subtype: TNBC	3 (11.1%)	8 (15.1%)	0.637
Subtype: HER-2 positive	3 (11.1%)	3 (5.7%)	
Technique: IMRT	10 (37.0%)	14 (26.4%)	0.439
Secondary EQD2 α/β=10, Gy	44.25 (42.29–44.25)	44.25 (44.25–50.00)	**<.001**

Values are median (IQR) or n (%).Secondary EQD2 distributions differed despite identical medians because whole-breast/chest wall reRT had a broader upper dose distribution.

Supplementary Table S4. Predictors of Locoregional recurrence-free survival (LRRFS) Following Breast Re-irradiation in multivariable Cox regression stratified by TNBC status.

Variable	Subgroups	Primary MV model		Sensitivity MV model	
		HR (95% CI)	p	HR (95% CI)	p
Recurrence timing	First	Ref		Ref	
	Second or subsequent	3.80 (1.34–10.73)	0.012	4.14 (1.25–13.72)	0.02
Grade	Early/Intermediate	Ref		Ref	
	High	3.09 (0.86–11.08)	0.08	3.17 (0.87–11.50)	0.08
Stage	Early	Ref		Ref	
	Locally advanced	4.99 (1.43–17.48)	0.012	5.11 (1.46–17.95)	0.01
	Metastatic	11.71 (2.60–52.62)	0.001	10.78 (2.16–53.80)	0.004
Dose at reirradiation	-			0.86 (0.30–2.49)	0.78

Supplementary Table S5. Descriptive details of CTCAE Grade toxicity distributions across domains.

Toxicity Domains	Gr I	Gr II	Gr III
Skin and Subcutaneous tissue	11 (9.7%)	35 (31%)	4 (3.5%)
Subcutaneous Fibrosis	6 (5.3%)	30 (26.5%)	1 (0.9%)
Breast pain/discomfort	2 (1.8%)	1 (0.9%)	0
Fat necrosis	3 (2.6%)	1 (0.9%)	0
Skin ulceration/ abscess	0	1 (0.9%)	2 (1.8%)
Soft tissue infection	0	0	1 (0.9%)
Rib and chest wall	19 (16.8%)	6 (5.3%)	1 (0.9%)
Shoulder dysfunction	4 (3.5%)	2 (1.8%)	0
Lymphedema	1 (0.9%)	2 (1.8%)	0
Pneumonitis	1 (0.9%)	5 (4.4%)	1 (0.9%)
Cardiomyopathy	0	1 (0.9%)	0

Distribution of toxicity can overlap between the individual domains.

Supplementary Table S6. Sensitivity analyses of Late Grade ≥2 Toxicity Following Breast Re-irradiation with EQD2 α/β=3

Variable	Subgroup	5-year incidence Gr ≥2 toxicity	P (Gray)	UV sHR	UV P	MV sHR	MV P
Interval between RT courses	>=98 months	26.6 (15.6-37.6)	0.022	Ref		Ref	
	<98 months	56.6 (38.4-74.8)		2.06 (1.13-3.74)	0.018	2.24 (0.96-5.21)	0.062
RT technique	3DCRT	43.4 (31.4-55.3)	0.004	Ref		Ref	
	IMRT	15.0 (1.1-28.9)		0.39 (0.21-0.72)	0.003	0.34 (0.16-0.71)	0.004
Dose per fraction at reirradiation	2 Gy	52.4 (30.9-73.9)	0.004	Ref		Ref	
	>2 Gy	55.8 (35.8-75.9)		0.90 (0.45-1.82)	0.773	1.87 (0.74-4.76)	0.188

	<2 Gy	15.3 (4.6-26.0)		0.32 (0.16-0.65)	0.002	0.73 (0.25-2.12)	0.562
Age at reirradiation	Per 10-year increase	-		0.70 (0.55-0.88)	0.003	0.87 (0.63-1.20)	0.386
Cumulative EQD2 alpha/beta=3	Per 10-Gy increase	-		2.57 (1.40-4.72)	0.002	2.33 (0.91-5.95)	0.077

Multivariable Cox models were stratified by TNBC status. Dermal LVI was excluded from the multivariable model because it was significantly correlated with stage (5%, 31%, and 40% in early, locally advanced, and metastatic disease, respectively; p<0.001), high grade (22% vs 5% in low/intermediate grade; p=0.007), and second/subsequent recurrence (31.3% vs 9.3% in first recurrence; p=0.013). The sensitivity model included secondary EQD2 α/β=10 per 10 Gy increase.

Supplementary Table S7. Predictors of Late Grade 3 Toxicity Following Breast Re-irradiation on Univariate Analysis

Variables	Subgroup	5-year incidence	P (Gray)	UV sHR	UV P
Interval between RT courses	≥98 months	3.0 (0.0–7.2)	0.970	Ref	
	<98 months	6.2 (0.0–14.6)		0.99 (0.17–5.84)	0.992
RT Technique	3DCRT	5.5 (0.2–10.8)	0.316	Ref	
	IMRT	0.0 (0.0–0.0)		0.37 (0.06–2.39)	0.296
Dose per fraction at reirradiation	2 Gy	4.2 (0.0–12.3)	0.851	Ref	
	>2 Gy	4.9 (0.0–14.4)		1.27 (0.15–10.43)	0.825
	<2 Gy	4.1 (0.0–9.8)		0.77 (0.12–4.96)	0.783
Prior Mastectomy	Yes	4.2 (0.0–10.0)	0.961	Ref	
	No	3.7 (0.0–8.9)		0.99 (0.20–4.85)	0.992
Age at reirradiation	Per 10-year increase	—		0.82 (0.40–1.66)	0.579
Cumulative EQD2	Per 10-Gy increase	—		4.32 (2.03–9.19)	<.001
